# The Association of Methotrexate, Sulfasalazine, and Hydroxychloroquine Use With Fracture in Postmenopausal Women With Rheumatoid Arthritis: Findings From the Women's Health Initiative

**DOI:** 10.1002/jbm4.10393

**Published:** 2020-08-18

**Authors:** Laura Carbone, Sowmya Vasan, Rachel Elam, Sandeepkumar Gupta, Omar Tolaymat, Carolyn Crandall, Jean Wactawski‐Wende, Karen C Johnson

**Affiliations:** ^1^ Department of Medicine, Division of Rheumatology, J. Harold Harrison MD, Distinguished University Chair in Rheumatology Medical College of Georgia at Augusta University Augusta GA USA; ^2^ Department of Rheumatology Charlie Norwood Veterans Affairs Medical Center Augusta GA USA; ^3^ Fred Hutchinson Cancer Research Center Seattle WA USA; ^4^ Department of Medicine, Division of Rheumatology Medical College of Georgia at Augusta University Augusta GA USA; ^5^ Department of Medicine, Division of General Internal Medicine and Health Services Research David Geffen School of Medicine at University of California, Los Angeles Los Angeles CA USA; ^6^ Department of Epidemiology and Environmental Health, School of Public Health and Health Professions University at Buffalo Buffalo NY USA; ^7^ Department of Preventive Medicine University of Tennessee Health Science Center Memphis TN USA

**Keywords:** COX PROPORTIONAL HAZARDS MODELING, DISEASE MODIFYING ANTIRHEUMATIC DRUGS, FRACTURE RISK ASSESSMENT, OSTEOPOROSIS, RHEUMATOID ARTHRITIS

## Abstract

This study was conducted to evaluate the extent to which disease‐modifying antirheumatic medications (DMARDs) used as part of a triple therapy for the treatment of rheumatoid arthritis (RA) including methotrexate, sulfasalazine, and hydroxychloroquine are associated with fractures in postmenopausal women with RA. Incident fractures following use of methotrexate, sulfasalazine, and/or hydroxychloroquine in postmenopausal women with RA in the Women's Health Initiative were estimated by Cox proportional hazards using hazard ratios (HRs) and 95% CIs after consideration of potential confounders. There were 1201 women with RA enrolled in the Women's Health Initiative included in these analyses, of which 74% were white, 17% were black, and 9% were of other or unknown race/ethnicity. Of the women with RA, 421 (35%) had not used methotrexate, sulfasalazine, or hydroxychloroquine, whereas 519 (43%) women had used methotrexate, 83 (7%) sulfasalazine, and 363 (30%) hydroxychloroquine alone or in combination at some time during study follow‐up. Over a median length of 6.46 years of follow‐up, in multivariable adjusted models, no statistically significant association was found between methotrexate (HR, 1.1; 95% CI, 0.8–1.6), sulfasalazine (HR, 0.6; 95% CI, 0.2–1.5), or hydroxychloroquine (HR, 1.0; 95% CI, 0.7–1.5) use and incident fractures or between combination therapy with methotrexate and sulfasalazine or methotrexate and hydroxychloroquine use (HR, 0.9; 95% CI, 0.5–1.6) and incident fractures. In conclusion, postmenopausal women with RA receiving any component of triple therapy should not be expected to have any substantial reduction in fracture risk from use of these DMARDs. © 2020 The Authors. *JBMR Plus* published by Wiley Periodicals LLC on behalf of American Society for Bone and Mineral Research.

## Introduction

Osteoporosis and osteoporotic‐related fractures are substantial comorbidities in patients with rheumatoid arthritis (RA). Women with RA have double the odds of osteoporosis diagnosed by DXA‐based BMD measurements of the femoral neck, total hip, and lumbar spine compared with those without RA.^(^ ^1^ ^)^ Rates of both hip and spine fractures are increased in those with RA, even in the absence of glucocorticoid use.^(^ ^2^ ^)^ Among the Women's Health Initiative (WHI) cohort, women with RA were found to have 1.5 times higher risk of fracture compared with women without any arthritis, a substantially higher increased risk than found for women with osteoarthritis.^(^ ^3^ ^)^ In recognition of the importance of RA as a risk factor for fracture, the widely used fracture risk assessment tool (FRAX) includes RA as an independent clinical risk factor for estimation of 10‐year risk of both hip and major osteoporotic fractures.^(^ ^4^ ^)^

Both traditional risk factors, as well as those common in persons with RA, including immobility, reduced physical activity, glucocorticoid use,^(^ ^5^, ^6^ ^)^ and falls,^(^ ^7^ ^)^ contribute to incident osteoporotic fractures. The increased fracture risk in patients with RA who are not taking glucocorticoids suggests that underlying inflammatory processes intrinsic to the disease state itself may contribute to fracture risk.^(^ ^8^ ^)^

It is increasingly appreciated that activities at the bone marrow level play a fundamental role in the development of osteoporosis, and the individual disease‐modifying antirheumatic medications (DMARDs) included in triple therapy—composed of methotrexate, sulfasalazine, and hydroxychloroquine—may affect this microenvironment. At high doses, methotrexate inhibits the proliferation of bone marrow stromal cell osteoblasts.^(^ ^9^ ^)^ However, at lower doses, in vitro studies suggest that methotrexate inhibits osteoclastogenesis and suppresses secretion of RANKL, which would be expected to be beneficial for osteoporosis.^(^ ^10^ ^)^ In the bone marrow, shifts in mesenchymal stem cells from osteogenic to adipogenic lineages are a fundamental mechanism underlying the pathogenesis of osteoporosis.^(^ ^11^ ^)^ Solute carrier family 7 member 11 (SLC7A11) is a cystine‐glutamate cotransporter that promotes an adipogenic pathway,^(^ ^12^ ^)^ and sulfasalazine inhibits SLC7A11.^(^ ^13^ ^)^ In preclinical models in ovariectomized mice, sulfasalazine decreased bone loss by shifting mesenchymal stem cells from an adipogenic to an osteoblastic pathway.^(^ ^11^ ^)^ Conversely, preclinical studies suggest that sulfasalazine may increase oxidative stress^(^ ^14^ ^)^ and ROS may contribute to osteoporosis. Hydroxychloroquine may also affect osteoblast and osteoclast formation. in vitro studies suggest that hydroxychloroquine both decreases mesenchymal‐derived osteoblast differentiation and inhibits the formation of osteoclasts.^(^ ^15^, ^16^ ^)^ These potential conflicting actions on the skeleton from methotrexate, sulfasalazine, and hydroxychloroquine use make it important to study the association of these medications with osteoporosis.

The advent of biologics has considerably revolutionized treatment for RA.^(^ ^17^ ^)^ However, combination therapy with traditional, nonbiologic DMARDs, including methotrexate, sulfasalazine, and hydroxychloroquine (triple therapy), remains an established treatment for RA after failure of methotrexate monotherapy and is considerably less expensive than biologics.^(^ ^18^, ^19^ ^)^

However, to our knowledge, there are no longitudinal reports of the association of methotrexate, sulfasalazine, and/or hydroxychloroquine alone or in combination with incident fractures in postmenopausal women with RA. The objective of this study was to utilize the WHI with its detailed assessments of medication use and incident fractures, to determine the association of methotrexate, sulfasalazine, and hydroxychloroquine use to incident osteoporotic fractures in postmenopausal women with RA.

## Materials and Methods

### Data source

Details of the original WHI recruitment, after which participants were followed for outcomes between 1993 and 2005, have been previously described.^(^ ^20^ ^)^ The WHI Study population includes women in the WHI observational study (OS) and clinical trials (CTs) (hormone therapy, dietary modification, and calcium and vitamin D trials). The WHI included postmenopausal women aged 50 to 79 years with estimated survival ≥3 years recruited between October 1, 1993 and December 31, 1998 at 40 clinical trial sites in the United States. At baseline, participants completed questionnaires regarding demographic characteristics, smoking, lifestyle, and medical history. Follow‐up questionnaires on medical history were collected semiannually in CT and annually in the OS participants through 2005. Protocols were approved by the institutional review board at each participating center. Women provided written informed consent for their participation in the original WHI study.

There were 161,808 women who were enrolled in the WHI, of which 93,676 were OS participants and 68,132 were CT participants. For the purposes of these analyses, all women who reported at baseline a history of RA based on self‐report plus having either a DMARD and/or a biologic use or presence of anticyclic citrullinated peptide (anti‐CCP) antibody from the WHI OS or CT were included. DMARD use for RA was defined as hydroxychloroquine, sulfasalazine, methotrexate, leflunomide, azathioprine, cyclosporine, gold, minocycline, and oral glucocorticosteroids based on a previous report in WHI.^(^ ^21^ ^)^ This prior report^(^ ^21^ ^)^ also defined cyclophosphamide as a DMARD for RA; however, this was not included in the current analyses as this medication is rarely used to treat RA alone. We also defined biologic use as any use of anakinra, adalimumab, infliximab, etanercept, or abatacept, as these medications were approved by the US Food and Drug Administration for use in RA by 2005, the final year of these analyses. We followed women from their WHI enrollment to the end of the main WHI Study in 2005.

#### *Incident fractures*

Specific outcomes and hospitalizations were assessed by questionnaires collected semiannually for the CT participants and annually for the OS participants through the end of the main WHI study. Fracture history was collected by self‐report. Hip fractures were also verified by review of medical records with central blinded adjudication.^(^ ^22^ ^)^ Fractures other than hip fractures were locally adjudicated for the CT participants and a subset of the OS participants (BMD participants) from 1993 to 2005. For the OS participants who did not have BMD measured, fracture information (other than hip fractures) was only obtained by self‐report with no adjudication.

Fracture subtype categories examined included: (i) hip fractures; (ii) upper limb fractures (humerus, radius, ulna, carpal, metacarpal, clavicle, or scapula—excluding fingers); (iii) lower limb fractures (femur [excluding hip], patella, tibia, fibula, tarsal or metatarsal—excluding toes); (iv) Central body fractures (hip, pelvis, or clinical spine); and (v) any clinical fracture (excluding fingers, toes, or coccyx); rib fractures were not collected in WHI.^(^ ^23^ ^)^ The median length of follow‐up was 6.50 years for hip fractures; 6.48 years for central body, upper and lower limb fractures; and 6.46 years for the category of all fractures.

### Methotrexate, sulfasalazine, and hydroxychloroquine use

Participants were asked about current prescription and over‐the‐counter medications taken in the past 2 weeks at baseline and years 1, 3, 6, and 9 of the main WHI study for CT participants and baseline and year 3 of the main WHI study for OS participants. Interviewers entered each medication into the database, which assigned drug codes using Medi‐Span software (First Data Bank, Inc., San Bruno, CA, USA). Information on duration of use was recorded. No information was collected on dose.

There were too few users of standard triple therapy (methotrexate, sulfasalazine, and hydroxychloroquine; *n* = 9) to determine the association of triple therapy use with incident fractures. Therefore, for the purposes of these analyses, we examined use of methotrexate and sulfasalazine, methotrexate and hydroxychloroquine (termed in this report “combination therapy”) and each of these DMARDs alone with incident fractures.

### Covariates

Height and weight at the baseline visit were measured in WHI as previously described^(^ ^24^ ^)^ and used to calculate BMI. Information on age, smoking status, prevalent fractures before WHI, general health, and physical activity were obtained by self‐report using questionnaires at the baseline visit.^(^ ^25^ ^)^ Weekly recreational physical activity and walking was calculated by multiplying an assigned energy expenditure level for each category of activity by the hours exercised per week to calculate total metabolic equivalents per week. History of two or more falls defined by self‐report of the number of times a participant fell and landed on the floor or ground in the preceding 12 months (participants were instructed not to include falls caused by sports activities, such as snow or water skiing or horseback riding) was included. History of diabetes was defined as a self‐report of physician diagnosis and treatment with insulin or oral antidiabetic drugs. Menopausal age was defined as the youngest age at which any of the following was experienced: last menstrual bleeding, removal of both ovaries, and/or beginning of hormone therapy. Alcohol use (beer, wine, liquor) was categorized to reflect overall consumption patterns and converted into a number of standard drinks/week and categorized (nondrinker versus current drinker; drinking categories: <1, 1 to <3, 3 to <7, ≥7 drinks/week). Enrollment in the OS or CT was recorded. Food frequency questionnaires were used to collect information on calcium and vitamin D intakes.^(^ ^26^ ^)^ Dietary and supplemental calcium and vitamin D were summed to derive total calcium and vitamin D intake.

At the baseline WHI examination, women were asked to report their joint pain severity (none, mild, moderate, severe). Anti‐CCP antibodies were measured in a subset of WHI women who reported RA at baseline or follow‐up.^(^ ^21^, ^27^ ^)^ DMARD and biologic use for RA, medications for treatment and/or prevention of osteoporosis (use of hormone therapy/estrogen [oral/patch] approved by the US Food and Drug Administration, raloxifene, teriparatide, bisphosphonates, calcitonin), and other medications that might affect bone metabolism, including proton pump inhibitors, thiazolidinediones, thyroid medications, psychoactive medications, antimanic drugs, hypnotics, anticonvulsants, antidepressants, and oral corticosteroids were included.

### Statistical analyses

Descriptive statistics were reported by combination therapy use at baseline. The mean and SD is presented for continuous variables and the frequencies and percentages for categorical variables. For continuous variables, two sample *t* tests were used to compare medication users with nonusers, whereas for categorical variables, Fisher's exact test was used to assess differences.

Cox proportional hazards models stratified by baseline age (10‐year intervals), study participation (CT or OS enrollment) were used to determine associations between use of combination therapy (methotrexate and sulfasalazine, methotrexate and hydroxychloroquine), and use of methotrexate, sulfasalazine, and hydroxychloroquine alone and incident fractures. Results are reported as hazard ratios (HRs) and 95% CIs. Change in any use of these medications over time was evaluated by entering current use as a time‐dependent exposure. Use at a particular follow‐up time was defined based on what was indicated on the participant's most recent medication inventory. The fracture HR at any follow‐up time was modeled as a function of the most recent medication use. Women contributed to follow‐up until the occurrence of the fracture outcome, death, or end of follow‐up (2005), whichever came first. To account for out‐of‐date medication use, women were censored if they did not have a medication inventory report within 3.5 years of their previous report, and they were not allowed to re‐enter back into the risk set if they had a medication inventory at a future date. We refined time of initiation of medication use from the duration of use variable reported at their medication inventory. This addresses change in medication use between medication inventory collections and extends participants recall of medication use to at most 1 or 2 years, and consequently does not supersede the preceding collection.^(^ ^28^ ^)^ Women in OS who had medication inventories done at unscheduled time points (*n* = 6) were further excluded from the above analyses.

An unadjusted model (model 1) and two multivariable adjusted models were fit for the fracture outcomes: a minimally adjusted model (model 2) adjusted only for baseline age, BMI, and race/ethnicity and a final adjusted model (model 3). This final model was limited to adjustment to five covariates, as this was the maximum number supported by the small number of fracture events. These five covariates included age, BMI, race/ethnicity, prevalent fracture, and other medication use that might affect bone metabolism (proton pump inhibitors, thiazolidinediones, thyroid medications, psychoactive medications, antimanic drugs, hypnotics, anticonvulsants, antidepressants, and oral corticosteroids). In an additional sensitivity analysis, we examined all other covariates listed above in a one‐factor‐at‐a‐time addition to our final model. Sensitivity analysis was indicated as these other covariates were a priori thought to be clinically important, despite a fully adjusted multivariate model not being statistically supported by the actual number of fracture events. Annualized percentage of fracture outcomes were calculated by use of methotrexate, sulfasalazine, and hydroxychloroquine alone and as part of combination therapy.

## Results

There were 1201 women with RA included in these analyses, of which 421 women (35%) had not used methotrexate, sulfasalazine, or hydroxychloroquine and 519 (43%) had used methotrexate, 83 (7%) sulfasalazine, and 363 (30%) hydroxychloroquine alone or in combination during the totality of the study follow‐up. There were 1061 women with RA (88%) who did not use combination therapy and 140 women with RA (12%) who used combination therapy (users of methotrexate and sulfasalazine or methotrexate and hydroxychloroquine) during the study follow‐up. Of the combination therapy users, 24 women (17%) were users of methotrexate and sulfasalazine and 126 (90%) were users of methotrexate and hydroxychloroquine during the study follow‐up.

At the baseline visit of WHI, compared with nonusers of methotrexate and sulfasalazine or methotrexate and hydroxychloroquine (*n* = 1109, 92%), combination therapy users (*n* = 92, 8%) were more likely to have a prevalent fracture (52% versus 40%, *p* = 0.03), more likely to be in the OS rather than the CT arms of the WHI study (86% versus 74%, *p* = 0.02), and less likely to have anti‐CCP antibody status measured at baseline (38% not measured versus 24%, *p* = 0.01). There were not significant differences between anti‐CCP positivity between combination therapy users and nonusers (45% versus 52%, *p* = 0.66) in those where anti‐CCP antibody was measured. No significant differences were found between age, BMI, race/ethnicity, smoking status, self‐reported health, physical activity level, history of two or more falls in the last 12 months, history of treated diabetes, age at menopause, alcohol use, joint pain severity, geographic study site enrollment, total calcium intake, total vitamin D intake, total caloric intake, conventional DMARD use, osteoporosis medication use, or other medication use affecting bone metabolism between combination therapy users and nonusers at baseline. No WHI enrollees with RA at baseline were using biologics. Among the CT arms, there was no difference in enrollment in the dietary modification trial or the hormone therapy trial between combination therapy users and nonusers (Table 1).

**Table 1:** Baseline Characteristics^a^ of Women With RA by Baseline Combination Therapy^b^ Use

	Combination therapy user (*n* = 92)	Combination therapy nonuser (*n* = 1109)	*p* Value^c^
Age at screening, years	63.37 ± 6.73	64.49 ± 7.11	0.15
BMI, kg/m^2^	27.01 ± 6.40	28.07 ± 6.20	0.12
Race/ethnicity			0.06
White	76 (82.61)	810 (73.04)	
Black	8 (8.70)	201 (18.12)	
Other/unknown	8 (8.70)	98 (8.84)	
Smoking			1.00
Never smoked	41 (44.57)	490 (44.75)	
Past smoker	43 (46.74)	509 (46.48)	
Current smoker	8 (8.70)	96 (8.77)	
Prevalent fracture at baseline	48 (52.17)	441 (40.16)	0.03
Self‐reported health status			0.32
Excellent	5 (5.49)	33 (3.01)	
Very good	17 (18.68)	241 (21.95)	
Good	49 (53.85)	504 (45.90)	
Fair	17 (18.68)	269 (24.50)	
Poor	3 (3.30)	51 (4.64)	
Physical activity, mets‐hours/week	9.68 ± 12.49	9.28 ± 10.97	0.74
History of 2 or more falls in last 12 months	12 (13.04)	173 (15.67)	0.65
History of treated diabetes	3 (3.26)	57 (5.14)	0.62
Age at menopause, years	47.83 ± 5.91	47.45 ± 7.09	0.57
Alcohol use			0.34
Nondrinker	13 (14.13)	132 (12.01)	
Past drinker	36 (39.13)	350 (31.85)	
Current drinker, <1 Drink per month or week	24 (26.09)	361 (32.85)	
Current drinker, 1–3 drinks/week	6 (6.52)	111 (10.10)	
Current drinker, 3–<7 drinks/week	9 (9.78)	74 (6.73)	
Current drinker, 7+ drinks/week	4 (4.35)	71 (6.46)	
CT or OS enrollment			0.02
OS	79 (85.87)	825 (74.39)	
CT	13 (14.13)	284 (25.61)	
Dietary modification (DM) trial enrollment			0.05
Not randomized to DM	84 (91.30)	899 (81.06)	
Placebo	5 (5.43)	127 (11.45)	
Treatment	3 (3.26)	83 (7.48)	
HT trial enrollment			0.75
Not randomized to HT	85 (92.39)	999 (90.08)	
Placebo (E, E + P Trial)	4 (4.35)	50 (4.51)	
Intervention (E, E + P Trial)	3 (3.26)	60 (5.41)	
Joint pain severity at baseline			0.05
None	0 (0)	52 (4.74)	
Mild	28 (30.43)	348 (31.69)	
Moderate	45 (48.91)	434 (39.53)	
Severe	19 (20.65)	264 (24.04)	
Anti‐CCP antibody status			0.01
Not measured	35 (38.04)	266 (23.99)	
Negative	16 (17.39)	267 (24.08)	
Positive	41 (44.57)	576 (51.94)	
Geographic study site			0.64
Northeast	19 (20.65)	250 (22.54)	
South	22 (23.91)	315 (28.40)	
Midwest	23 (25.00)	265 (23.90)	
West	28 (30.43)	279 (25.16)	
Log transformed total calcium intake (diet plus supplements)	7.03 ± 0.59	6.94 ± 0.65	0.18
Log transformed total vitamin D intake (diet plus supplements)	5.77 ± 0.79	5.66 ± 0.90	0.25
Total caloric intake, calories	7.26 ± 0.39	7.25 ± 0.45	0.92
DMARD (excluding combination therapy) use	42 (45.65)	411 (37.06)	0.12
Minocycline	1 (1.09)	18 (1.62)	1.00
Leflunomide	0 (0)	0 (0)	
Azathioprine	5 (5.43)	28 (2.52)	0.10
Cyclosporine	0 (0)	4 (0.36)	1.00
Gold	0 (0)	10 (0.90)	1.00
Glucocorticosteroids	38 (41.30)	372 (33.54)	0.14
Osteoporosis medication use^d^	49 (53.26)	491 (44.27)	0.10
Other medication use^e^	39 (42.39)	543 (48.96)	0.23

Anti‐CCP = Anti‐cyclic citrullinated protein antibody; BMI = body mass index (calculated as weight in kilograms divided by height in meters squared); CT = clinical trial; DMARD = disease‐modifying antirheumatic drug; E = estrogen‐alone; E + P = estrogen plus progestin; HT = hormone therapy; Met‐hours/week = metabolic equivalents‐hours/week; OS = observational study.

a Values expressed and mean ± SD for continuous variables and *N* (%) for categorical variables.

b Combination therapy was defined as use of methotrexate and hydroxychloroquine, or methotrexate and sulfasalazine.

c Differences were assessed using *T* tests for continuous variables and Fisher's exact test for categorical variables.

d Use of hormone therapy/estrogen (oral/patch), raloxifene, teriparatide, bisphosphonates, and calcitonin.

e Use of other medications affecting bone metabolism: proton pump inhibitors, thiazolidinediones, thyroid medications, psychoactive medications, antimanic drugs, hypnotics, anticonvulsants, antidepressants, and oral corticosteroids.

Among methotrexate users, the annualized fracture incidence was 2.28% compared with 2.08% in nonusers, a difference that was not significant (*p* = 0.55). In unadjusted models, no statistically significant association was found between methotrexate use and all fractures (HR, 1.2; 95% CI, 0.8–1.6) or any fracture subtype category examined (Table 2). In minimally adjusted (HR, 1.1; 95% CI, 0.8–1.5) and final (HR, 1.1; 95% CI, 0.8–1.6) models, no statistically significant association was found between methotrexate use and all fractures, or similarly among any fracture subtype category examined (Table 2). Among sulfasalazine users, the annualized fracture incidence was 2.89% compared with 2.11% in nonusers, a difference that was not significant (*p* = 0.35). In unadjusted (HR, 0.6; 95% CI, 0.2–1.6), minimally adjusted (HR, 0.6; 95% CI, 0.2–1.5), and final (HR, 0.6; 95% CI, 0.2–1.5) models, no statistically significant association was found between sulfasalazine use and all fractures (Table 3). All three models for central body fractures by sulfasalazine use similarly yielded no significant associations (Table 3). By Fisher's exact test, a significantly higher annualize fracture incidence of upper limb fractures was seen in sulfasalazine users (1.51%) compared with nonusers (0.54%, *p* = 0.03). However, in unadjusted (HR, 1.9; 95% CI, 0.6–6.2), minimally adjusted (HR, 1.8; 95% CI, 0.5–5.9), and final (HR, 1.8; 95% CI, 0.5–6.0) models, the association between sulfasalazine use and upper limb fractures was no longer significant (Table 3). There were too few lower limb and hip fractures among sulfasalazine users to test the association between sulfasalazine use and these fracture subtypes. Among hydroxychloroquine users, the annualized fracture incidence was 2.39% compared with 2.06% in nonusers, a difference that was not significant (*p* = 0.40). In unadjusted (HR, 1.1; 95% CI, 0.8–1.7), minimally adjusted (HR, 1.1; 95% CI, 0.7–1.5), and final (HR, 1.0; 95% CI, 0.7–1.5) models, no statistically significant association was found between hydroxychloroquine use and all fractures, or similarly among any fracture subtype category examined (Table 4).

**Table 2:** Incident Fractures^a^ by Use (*n* = 407) or Nonuse (*n* = 788) of Methotrexate Reported as Hazard Ratio (95% CI)^b^

	No.	Annualized, %	*p* Value^c^	Model 1^d^	Model 2^e^	Model 3^f^
All fractures^g^						
Nonuser	111	2.08	0.55	1 (Reference)	1 (Reference)	1 (Reference)
User	63	2.28		1.2 (0.8–1.6)	1.1 (0.8–1.5)	1.1 (0.8–1.6)
Central body fractures^h^						
Nonuser	54	0.97	1.00	1 (Reference)	1 (Reference)	1 (Reference)
User	28	0.97		1.2 (0.7–2.0)	1.1 (0.7–1.9)	1.3 (0.7–1.9)
Upper limb fractures^i^						
Nonuser	30	0.53	0.36	1 (Reference)	1 (Reference)	1 (Reference)
User	20	0.69		1.2 (0.6–2.3)	1.2 (0.6–2.3)	1.1 (0.6–2.2)
Lower limb fractures^j^						
Nonuser	41	0.73	0.79	1 (Reference)	1 (Reference)	1 (Reference)
User	23	0.80		1.0 (0.6–1.8)	1.0 (0.6–1.7)	1.0 (0.6–1.7)
Hip fractures						
Nonuser	28	0.48	0.53	1 (Reference)	1 (Reference)	1 (Reference)
User	18	0.60		1.6 (0.8–3.2)	1.5 (0.7–3.0)	1.5 (0.7–3.0)

a Fractures (adjudicated or self‐report) reported until the end of the main Women's Health Initiative study are used.

b All proportional hazards models are stratified by age at baseline (10‐year interval) and study participation (clinical trials or observational study enrollment). Methotrexate use entered the model as a time‐dependent variable.

c Fisher's exact test.

d Unadjusted model.

e Adjusted for: Age, BMI, race/ethnicity.

f Adjusted for: Age, BMI, race/ethnicity, prevalent fracture, baseline use of other medication affecting bone metabolism (proton pump inhibitors, thiazolidinediones, thyroid medications, psychoactive medications, antimanic drugs, hypnotics, anticonvulsants, antidepressants, and oral corticosteroids).

g First type of fracture (if multiple types occurred) was captured and time to that first fracture was modeled (excluding fingers, toes, and coccyx).a

h Defined as the first occurrence of hip, pelvis, or spine fracture.

i Defined as the first occurrence of humerus, radius, ulna, carpal, metacarpal clavicle, or scapula—excluding fingers.

j Defined as the first occurrence of femur (excluding hip), patella, tibia, fibula, tarsal or metatarsal—excluding toes.

**Table 3:** Incident Fractures^a^ by Use (*n* = 59) or Nonuse (*n* = 1,136) of Sulfasalazine Reported as Hazard Ratio (95% CI)^b^

	No.	Annualized, %	*p* Value^c^	Model 1^d^	Model 2^e^	Model 3^f^
All fractures^g^						
Nonuser	163	2.11	0.35	1 (Reference)	1 (Reference)	1 (Reference)
User	11	2.89		0.6 (0.2–1.6)	0.6 (0.2–1.5)	0.6 (0.2–1.5)
Central body fractures^h^						
Nonuser	78	0.97	1.00	1 (Reference)	1 (Reference)	1 (Reference)
User	4	1.00		0.4 (0.1–3.0)	0.4 (0.1–2.8)	0.4 (0.1–3.1)
Upper limb fractures^i^						
Nonuser	44	0.54	0.03	1 (Reference)	1 (Reference)	1 (Reference)
User	6	1.51		1.9 (0.6–6.2)	1.8 (0.5–5.9)	1.8 (0.5–6.0)

a Fractures (adjudicated or self‐report) reported until the end of the main Women's Health Initiative study are used.

b All proportional hazards models are stratified by age at baseline (10‐year interval) and study participation (clinical trials or observational study enrollment). Sulfasalazine use entered the model as a time‐dependent variable.

c Fisher's exact test.

d Unadjusted model.

e Adjusted for: Age, BMI, race/ethnicity.

f Adjusted for: Age, BMI, race/ethnicity, prevalent fracture, baseline use of other medication affecting bone metabolism (proton pump inhibitors, thiazolidinediones, thyroid medications, psychoactive medications, antimanic drugs, hypnotics, anticonvulsants, antidepressants, and oral corticosteroids).

g First type of fracture (if multiple types occurred) was captured and time to that first fracture was modeled (excluding fingers, toes, and coccyx).

h Defined as the first occurrence of hip, pelvis, or spine fracture.

i Defined as the first occurrence of humerus, radius, ulna, carpal, metacarpal clavicle, or scapula—excluding fingers.

**Table 4:** Incident Fractures^a^ by Use (*n* = 299) or Nonuse (*n* = 896) of Hydroxychloroquine Reported as Hazard Ratio (95% CI)^b^

	No.	Annualized, %	*p* Value^c^	Model 1^d^	Model 2^e^	Model 3^f^
All fractures^g^						
Nonuser	126	2.06	0.40	1 (Reference)	1 (Reference)	1 (Reference)
User	48	2.39		1.1 (0.8–1.7)	1.1 (0.7–1.5)	1.0 (0.7–1.5)
Central body fractures^h^						
Nonuser	55	0.86	0.11	1 (Reference)	1 (Reference)	1 (Reference)
User	27	1.30		1.2 (0.7–2.1)	1.2 (0.6–2.1)	1.2 (0.6–2.1)
Upper limb fractures^i^						
Nonuser	42	0.66	0.18	1 (Reference)	1 (Reference)	1 (Reference)
User	8	0.38		0.5 (0.2–1.4)	0.5 (0.2–1.3)	0.5 (0.2–1.3)
Lower limb fractures^j^						
Nonuser	45	0.70		1 (Reference)	1 (Reference)	1 (Reference)
User	19	0.91	0.38	1.3 (0.7–2.3)	1.2 (0.6–2.1)	1.2 (0.6–2.1)
Hip fractures						
Nonuser	35	0.52	1.00	1 (Reference)	1 (Reference)	1 (Reference)
User	11	0.50		0.7 (0.3–1.8)	0.7 (0.3–1.7)	0.7 (0.3–1.8)

a Fractures (adjudicated or self‐report) reported until the end of the main Women's Health Initiative study are used.

b All proportional hazards models are stratified by age at baseline (10‐year interval) and study participation (clinical trials or observational study enrollment). Hydroxychloroquine use entered the model as a time‐dependent variable.

c Fisher's exact test.

d Unadjusted model.

e Adjusted for: Age, BMI, race/ethnicity.

f Adjusted for: Age, BMI, race/ethnicity, prevalent fracture, baseline use of other medication affecting bone metabolism (proton pump inhibitors, thiazolidinediones, thyroid medications, psychoactive medications, antimanic drugs, hypnotics, anticonvulsants, antidepressants, and oral corticosteroids).

g First type of fracture (if multiple types occurred) was captured and time to that first fracture was modeled (excluding fingers, toes, and coccyx).

h Defined as the first occurrence of hip, pelvis, or spine fracture.

i Defined as the first occurrence of humerus, radius, ulna, carpal, metacarpal clavicle, or scapula—excluding fingers.

j Defined as the first occurrence of femur (excluding hip), patella, tibia, fibula, tarsal or metatarsal—excluding toes.

Among users of combination therapy, the annualized fracture incidence was 2.09% compared with 2.15% in nonusers, a difference that was not significant (*p* = 1.0). In unadjusted (HR, 0.9; 95% CI, 0.5–1.8), minimally adjusted (HR, 0.9; 95% CI, 0.5–1.6), and final (HR, 0.9; 95% CI, 0.5–1.6) models, there was no significant association between combination therapy use and all fractures (Table 5). Similarly, there was no significant association between combination therapy use and any fracture subtype category examined (Table 5).

**Table 5:** Incident Fractures^a^ by Use (*n* = 92) or Nonuse (*n* = 1103) of Combination Therapy^b^ Reported as Hazard Ratio (95% CI)^c^

	No.	Annualized, %	*p* Value^d^	Model 1^e^	Model 2^f^	Model 3^g^
All fractures^h^						
Nonuser	161	2.15	1.00	1 (Reference)	1 (Reference)	1 (Reference)
User	13	2.09		0.9 (0.5–1.8)	0.9 (0.5–1.6)	0.9 (0.5–1.6)
Central body fractures^i^						
Nonuser	75	0.96	0.67	1 (Reference)	1 (Reference)	1 (Reference)
User	7	1.08		1.2 (0.5–3.0)	1.1 (0.4–2.8)	1.1 (0.5–2.9)
Upper limb fractures^j^						
Nonuser	47	0.60	1.00	1 (Reference)	1 (Reference)	1 (Reference)
User	3	0.46		0.3 (0.0–2.3)	0.3 (0.0–2.2)	0.3 (0.0–2.1)
Lower limb fractures^k^						
Nonuser	59	0.75	1.00	1 (Reference)	1 (Reference)	1 (Reference)
User	5	0.78		1.1 (0.4–2.7)	1.0 (0.4–2.6)	1.0 (0.4–2.6)
Hip fractures						
Nonuser	43	0.52	1.00	1 (Reference)	1 (Reference)	1 (Reference)
User	3	0.44		0.4 (0.1–2.9)	0.4 (0.1–2.9)	0.4 (0.1–3.1)

a Fractures (adjudicated or self‐report) reported until the end of the main Women's Health Initiative study are used.

b Combination therapy was defined as use of methotrexate and hydroxychloroquine, or methotrexate and sulfasalazine.

c All proportional hazards models are stratified by age at baseline (10‐year interval) and study participation (clinical trials or observational study enrollment). Combination therapy use entered the model as a time‐dependent variable.

d Fisher's exact test.

e Unadjusted model.

f Adjusted for: Age, BMI, race/ethnicity.

g Adjusted for: Age, BMI, race/ethnicity, prevalent fracture, baseline use of other medication affecting bone metabolism (proton pump inhibitors, thiazolidinediones, thyroid medications, psychoactive medications, antimanic drugs, hypnotics, anticonvulsants, antidepressants, and oral corticosteroids).

h First type of fracture (if multiple types occurred) was captured and time to that first fracture was modeled (excluding fingers, toes, and coccyx).

i Defined as the first occurrence of hip, pelvis, or spine fracture.

j Defined as the first occurrence of humerus, radius, ulna, carpal, metacarpal clavicle, or scapula—excluding fingers.

k Defined as the first occurrence of femur (excluding hip), patella, tibia, fibula, tarsal or metatarsal—excluding toes.

Sensitivity models, including all other covariates thought a priori to be clinically significant, but not included in the final model, did not change the significance of study findings with respect to use of methotrexate, sulfasalazine, or hydroxychloroquine alone or in combination therapy relative to all fractures or any fracture subtype category (data not shown).

## Discussion

In postmenopausal women with RA included in the WHI, after adjustment for potential confounding variables, no significant association was found between use of methotrexate, sulfasalazine, or hydroxychloroquine alone or in combination with overall or site‐specific fractures, including fractures of the central body, upper limb, lower limb, and hip.

There are no prior longitudinal population‐based studies of the association of methotrexate use with incident fractures in patients with RA. In contrast with our findings of no association of methotrexate use with incident fractures, a few case reports have suggested that use of methotrexate was felt to have caused a fracture, so‐called methotrexate osteopathy.^(^ ^29^, ^30^, ^31^ ^)^ To our knowledge, there are no prior reports implicating sulfasalazine or hydroxychloroquine use in patients with RA with incident fractures.

BMD is a predictor of fractures in RA,^(^ ^32^ ^)^ and bone turnover markers predict fractures in elderly women.^(^ ^33^ ^)^ The number of women with RA who had BMD measurements in WHI was too small to determine the association of methotrexate, sulfasalazine, and/or hydroxychloroquine use with BMD. However, our findings of no association between any of these medications alone or in combination with incident fractures are in accord with previous reports suggesting no substantial benefit for methotrexate^(^ ^34^, ^35^, ^36^, ^37^, ^38^, ^39^, ^40^, ^41^ ^)^ or sulfasalazine or hydroxychloroquine in combination with methotrexate on BMD.^(^ ^42^ ^)^ A recent study including 368 Taiwanese patients with RA, reported that use of conventional DMARDs including sulfasalazine, methotrexate, hydroxychloroquine for 3 years did not prevent decreases in BMD at the lumbar spine and hip.^(^ ^43^ ^)^ In contrast, in one report including Swedish men with RA, there was a statistically significant positive correlation between patients treated with sulfasalazine and BMD at the trochanter, with sulfasalazine‐treated patients demonstrating 8% to 10% higher BMD than those on no therapy, corticosteroids alone, or other DMARDs (methotrexate, cyclosporine A, or injectable gold).^(^ ^44^ ^)^

Previous reports of the effects of sulfasalazine and hydroxychloroquine on bone turnover are conflicting. Our findings of no association of sulfasalazine or hydroxychloroquine use with fractures are in contrast with reports that these drugs might reduce bone resorption.^(^ ^16^, ^45^ ^)^ In support of our findings, relative to triple therapy in RA with methotrexate, sulfasalazine, and hydroxychloroquine, in one small randomized study there were no significant differences in changes in bone turnover at the lumbar spine or femoral neck in those assigned to triple therapy compared with treatment with infliximab plus methotrexate.^(^ ^46^ ^)^

That few women with RA were taking sulfasalazine in WHI (approximately 8% reported combination therapy use, and only 5% reported sulfasalazine use alone) is in accordance with previous reports. Patients with RA are more likely to be persistent and adherent to combination therapy with a tumor necrosis factor alpha inhibitor and methotrexate than combination therapy with methotrexate, sulfasalazine, and hydroxychloroquine.^(^ ^47^, ^48^ ^)^

There are a number of limitations to these analyses. To start, this was a post hoc observational study. The number of women with RA in WHI was small, with few fractures, and our power to detect differences in fractures by treatment of RA was limited. Although we adjusted for known covariates of importance to the extent that the actual number of fracture events allowed and performed a sensitivity analysis for other available covariates thought a priori to be clinically significant without changes in the study findings, residual confounding remains a possibility. We lacked measurements of renal function and serum vitamin D levels. We did not have measures of disease activity or severity in RA, including inflammatory markers, imaging with erosive disease determined, or duration of RA. Further, we did not have information on the doses of medications used, or in fact, whether the medications were taken as directed. Anti‐CCP antibodies were not measured in all women, and they were less likely to be measured in the combination therapy user group compared with nonusers. We chose a case definition of RA for our study to include all women with a self‐reported history of RA plus either DMARD and/or biologic use or presence of anti‐CCP antibody. Use of this case definition of RA in the WHI cohort has been validated in the literature,^(^ ^49^ ^)^ but some misclassification of RA diagnosis likely occurred. Therapy for RA has changed in recent years, and our data set did not include women on biologics to determine the association of these medications with fracture risk. The validity of self‐report of fractures in WHI varies by the fracture site, but the average agreement confirmed by medical records for a single fracture site is 71%.^(^ ^50^ ^)^ Finally, our findings were from a population of postmenopausal women enrolled in WHI, and the results may not be generalizable to other populations.

Our study also has strengths. This study was conducted using a large, well‐characterized, population‐based study of postmenopausal women. To our knowledge, this is the first study to evaluate incident fracture risk in a large cohort of postmenopausal women with RA treated with any medication included as part of triple therapy.

In conclusion, use of methotrexate, sulfasalazine, or hydroxychloroquine alone or in combination is not associated with incident fractures in postmenopausal women with RA. Postmenopausal women with RA receiving any component of triple therapy should not be expected to have any substantial reduction in osteoporotic risk from use of these medications.

## Disclosures

All authors declare no conflicts of interest.

## Author Contributions

**Laura Carbone:** Conceptualization; data curation; formal analysis; investigation; methodology; supervision; validation; visualization; writing‐original draft; writing‐review and editing. **Sowmya Vasan:** Data curation; formal analysis; methodology; writing‐original draft; writing‐review and editing. **Rachel Elam:** Investigation; validation; writing‐original draft; writing‐review and editing. **Sandeepkumar Gupta:** Writing‐original draft; writing‐review and editing. **Omar Tolaymat:** Writing‐original draft; writing‐review and editing. **Carolyn Crandall:** Conceptualization; investigation; methodology; validation; visualization; writing‐original draft; writing‐review and editing. **Jean Wactawski‐Wende:** Conceptualization; investigation; methodology; validation; visualization; writing‐original draft; writing‐review and editing. **Karen Johnson:** Conceptualization; investigation; methodology; validation; visualization; writing‐original draft; writing‐review and editing.

### Peer Review

The peer review history for this article is available at https://publons.com/publon/10.1002/jbm4.10393.
